# Electrochemical sensors based on sewage sludge–derived biochar for the analysis of anthocyanins in berry fruits

**DOI:** 10.1007/s00216-022-04062-y

**Published:** 2022-04-26

**Authors:** Patrick Severin Sfragano, Serena Laschi, Lapo Renai, Michelangelo Fichera, Massimo Del Bubba, Ilaria Palchetti

**Affiliations:** Department of Chemistry Ugo Schiff, University of Florence, Via della Lastruccia 3-13, 50019 Sesto Fiorentino, Italy

**Keywords:** Sewage sludge, Biochar, Sensor, Electrochemical, Anthocyanins

## Abstract

The reutilization of waste and the reduction of the general environmental impact of every production are fundamental goals that must be achieved in the framework of a circular economy. Recycled carbon-rich materials may represent a promising alternative to other less-sustainable carbonaceous materials used in the production of electrochemical sensing platforms. Herein, we propose an innovative carbon paste electrode (CPE) composed of biochar derived from biological sludge obtained from municipal and industrial wastewater treatment plants. The physicochemical properties of the biochar after a chemical treatment with an acidic solution obtained from industrial by-products were investigated. The electrode surface characterization was carried out by analyzing common redox probes and multiple phenols bearing varying numbers of –OH and –OCH_3_ groups in their structure. Furthermore, the CPE was also tested on the evaluation of the phenolic fingerprints of *Vaccinium myrtillus*, *Vaccinium uliginosum* subsp. *gaultherioides*, and *Fragaria* × *ananassa*. Standard anthocyanin mixtures and extracts of the aforementioned fruits were analyzed to provide a phenolic characterization of real samples. The obtained results show that the sewage sludge–derived biochar can be a promising material for the development of electroanalytical sensors.

**Graphical abstract:**

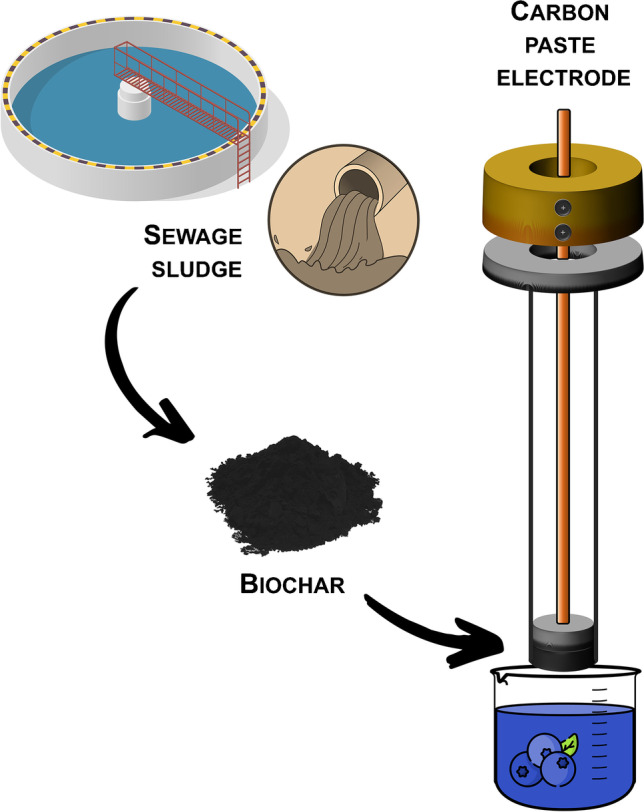

**Supplementary Information:**

The online version contains supplementary material available at 10.1007/s00216-022-04062-y.

## Introduction

Recycled carbonaceous materials (RCM) obtained from the pyrolysis or gasification of waste biomass are recently receiving increasing attention for use in different areas of analytical chemistry [1]. In view of the similarities between some RCMs (e.g., biochar) and carbon black (CB), the use of RCM represents a cost-effective alternative to other carbon-based materials in electrochemical (bio)sensing and energetics [1–3]. CB is a nanomaterial made up of amorphous carbon, containing minimal amounts of oxygen, nitrogen, and hydrogen. Depending on the final application, CB can be produced by partial combustion or thermal decomposition of a hydrocarbon fuel with a limited supply of combustion air at high temperatures [4]. CB is the main commercially marketed form of carbon nanostructured material, widely used in electrochemical sensing applications [5, 6]. In this field, where more complex carbon-based materials, such as carbon nanotubes or graphene [7–10], are widely used with excellent results, CB has recently been employed for its versatility [11]. The use of RCMs as an alternative to virgin materials would be a step forward in the development of more sustainable electrochemical sensors [12].

Biochar is a type of RCM obtained by controlled pyrolysis or gasification of waste biomass, the physicochemical properties of which strongly vary depending on the feedstock and the thermo-chemical conditions used [13, 14]. Accordingly, materials with different specific surface areas, porosities, structures, and chemical compositions can be obtained. A wide range of waste biomasses, of both animal and vegetal origins, have been used so far for biochar production, including agricultural wastes [15, 16], wood residues [17], manure [18], and biological sludge [19]. However, to the best of our knowledge, biochar used for electroanalytical applications is derived from a narrower group of waste biomasses, them being limited to few vegetal wastes, such as castor oil, wheat straw, residues of forest cuttings, spent grain, spent coffee grounds, and others [20–25].

The production and application of electroanalytical sensors based on biochar from sewage sludge is a topic that has not yet been explored. In this regard, it should be stressed that the recycling of biological sludge from wastewater treatment plants (WWTPs) represents an attractive option. In fact, biological sludge is a widely available waste material of high environmental concern, the disposal of which involves high costs, whereas its reuse represents a win–win solution, in line with a modern approach toward waste management and circular economy [12]. However, the use of biochar from sewage sludge entails a series of difficulties related to the limited specific surface area as well as the presence of metal species and other inorganic compounds [13, 26]. To solve these problems, biochar may undergo various treatments, such as physical and chemical activation, the latter generally based on its impregnation with acids or bases, aiming at increasing surface area, modulating porosity, introducing surface functional groups, and removing unwanted materials and compounds. However, these treatments create additional costs and reduce the sustainability of the process, as virgin reagents are commonly used for this purpose. In this context, the use of chemical treatments based on low-cost recycled products, instead of virgin ones, would represent an interesting approach for the improvement of biochar properties.

On the basis of the aforementioned considerations, in this work, we have investigated the possibility of fabricating electrochemical sensors based on biochar derived from biological sludge and their application for the determination of antioxidant compounds in agri-food samples. Thus, two types of carbon paste electrodes (CPEs) were prepared, mixing mineral oil and graphite with sewage-sludge-biochar (SSB) from a WWTP collecting mixed municipal-industrial wastewater. In detail, the CPEs were fabricated using a biochar prepared by the pyrolysis of a mixture of sewage sludge and woody waste from forest cuttings, untreated and treated by washing with an acidic solution derived from the gasification of woody wastes. The electrochemical characterization of the SSB-CPEs was carried out by cyclic voltammetry and electrochemical impedance spectroscopy with ferro/ferricyanide as redox indicator. The CPEs were successively tested in cyclic voltammetry and differential pulse voltammetry toward different phenolic compounds, including vanillin, hydroquinone, catechol, resorcinol, and gallic acid. These compounds were chosen since they are frequently used as redox systems for evaluating the electrode surface condition [27, 28]. Moreover, phenolic compounds have antioxidant properties, emphasizing the importance of their determination in food samples. The SSB-CPEs were then challenged for monitoring the phenolic fingerprints of *Vaccinium myrtillus*, *Vaccinium uliginosum* subsp*. gaultherioides*, and *Fragaria* × *ananassa* berries, which were selected based on their different (poly)phenolic compositions, particularly of the anthocyanin fraction [29, 30]. Furthermore, *V. myrtillus* and *F.* × *ananassa* berries are widely recognized as functional foods and commonly consumed as fresh and processed products [31]. Last but not least, *V. myrtillus* (i.e., bilberry) and *V. uliginosum* subsp*. gaultherioides* (often referred to as “false bilberry”) are wild berries with similar phenotypes and growing in the same zones, but providing very different sensorial properties, the latter characterized by much worse taste and odor attributes [32]. The comparative evaluation of their anthocyanin fraction through rapid electrochemical screening is therefore interesting. Accordingly, several standard anthocyanins were individually tested, and their electrochemical behavior was evaluated by square-wave voltammetry. Standard anthocyanin mixtures, simulating their relative abundance in the aforementioned fruits and berry extracts, were also analyzed to verify the applicability of the SSB-CPE to the screening of real samples.

## Materials and methods

### Reagents

Graphite powder, mineral oil, hexacyanoferrate (II)/(III) (K_4_[Fe(CN)_6_]/K_3_[Fe(CN)_6_]), potassium chloride (KCl), hydroquinone, catechol, resorcinol, gallic acid, vanillin, sodium dihydrogen phosphate (NaH_2_PO_4_ · 2H_2_O), sodium hydrogen phosphate (Na_2_HPO_4_ · 2H_2_O), acetic acid (CH_3_COOH), and sodium acetate anhydrous (CH_3_COONa) were analytical grade and were purchased from Sigma-Aldrich (Milan, Italy). BioDea acidic solution (a liquid by-product of the gasification of woody waste) was provided by Romana Maceri Centro Italia S.r.l. (Civitella in Val di Chiana, Italy). The anthocyanins delphinidin-3-*O*-glucoside chloride (myrtillin chloride), peonidin-3-*O*-arabinoside chloride, peonidin-3-*O*-glucoside chloride, cyanidin-3-*O*-galactoside chloride, cyanidin-3-*O*-glucoside chloride (kuromanin chloride), petunidin-3-*O*-glucoside chloride, malvidin-3,5-di-*O*-glucoside chloride, malvidin-3-*O*-glucoside chloride (oenin chloride), and pelargonidin-3-*O*-rutinoside chloride were from Extrasynthese (Geneve, France).

Water solutions were prepared with deionized water obtained with a Milli-Q water purification system (Milan, Italy).

### Biochar preparation and treatment

Biochar was produced via pyrolysis of a 70/30 (w/w) mixture of waste woody biomass (WWB, mainly oak) and biological sludge (BS) in a muffle furnace (Gefran 1001, Vittadini Strumentazione, Milan, Italy), properly modified to allow the heating process in N_2_-saturated conditions. Both feedstocks were dried at 105 °C overnight prior to use. The pyrolysis was carried out from room temperature to 850 °C with an average increase of 10 °C min^−1^ and a final isotherm at 850 °C for 60 min. The WWB and the BS used as feedstocks for the production of biochar were supplied by Romana Maceri Centro Italia S.r.l. (Civitella in Val di Chiana, Italy) and Gestione Impianti di Depurazione Acque S.p.A. (Prato, Italy), respectively. The biochar was washed with deionized water (1 g in 30 mL of water under stirring for 30 min), dried at 105 °C overnight, and then used for the fabrication of the electrodes. A portion of the biochar thus obtained was subjected to a further process (patent pending) consisting in its washing with the BioDea solution, a commercially available acidic liquid by-product of the gasification of woody waste, and deionized water. The two biochars are identified below as SSB-U (untreated biochar) and SSB-T (washing-treated biochar), respectively.

### Biochar-based electrode fabrication

Modified carbon pastes were prepared with proportions of 10% (w/w) mineral oil, considering 90% (w/w) biochar and 10% (w/w) graphite powder. Graphite powder with an average particle diameter ≤ 20 μm was added as electroactive graphitic component, in order to improve the overall conductivity and the performance of the electrode.

The components were homogenized with a three-roll mill, and the pastes were compacted in a PVC tube support, containing a nickel-plated copper rod as electric contact. The ready-to-use electrodes had dimensions of 10 cm in length and 3.5 mm in diameter. The electrode surface is 3.0 mm in diameter. The ohmic resistance varies in terms of 65–70 Ω for each CPE.

Two different types of CPEs were prepared; one was fabricated by using an untreated biochar (SSB-U CPE) and the other was made by using the washing-treated biochar (SSB-T CPE).

The role of the mass of active material [33] was not evaluated in the present work.

### Electrochemical measurements

Electrochemical measurements were performed with an Autolab PGSTAT12 computerized electrochemical system equipped with the FRA2 frequency response analyzer and controlled by NOVA software (Metrohm). A Pt wire, an Ag/AgCl (saturated KCl) electrode, and a SSB-CPE served as the auxiliary, reference, and working electrode, respectively.

Cyclic voltammetry (CV) measurements were performed at a scan rate of 0.025 V s^−1^ in the potential range from −0.5 to +1 V, unless otherwise stated. Electrochemical impedance spectroscopy (EIS) measurements were performed with a sinusoidal voltage of 10 mV amplitude, at an OCP value (vs*.* Ag/AgCl) in a frequency range of 100 kHz to 10 mHz. The EIS spectra were plotted as the complex plane diagrams (Nyquist plots). Differential pulse voltammetry (DPV) conditions were as follows: pulse amplitude, 90 mV; pulse width, 60 ms; and scan rate, 30 mV s^−1^. DPV voltammograms underwent a baseline correction with a moving average filter, and the data obtained were smoothed with a 2^nd^-order Savitzky-Golay filter, using NOVA’s built-in tool. Square-wave voltammetry (SWV) conditions were as follows: frequency (*f*) of 25 Hz, square-wave amplitude (Esw) of 50 mV, and step potential (Es) of 2 mV. The scan was performed in the potential range from 0.0 to +1.0 V (vs*.* Ag/AgCl).

All measurements were performed at room temperature. After the voltammetric measurements, the electrode surface was subjected to a simple cleaning step by gently scrubbing the paste in circular motions on absorbent lab paper, allowing the removal of impurities on the electrode surface and multiple reuses of the electrode.

### Physicochemical characterization of biochars

The textural properties of the biochars were evaluated by nitrogen adsorption and desorption experiments at −196 °C using a Porosity Analyzer Micromeritics (Norcross, GA, USA) model ASAP 2020. The surface area was calculated by using the Brunauer-Emmet-Teller (BET) and the Langmuir methods applied to nitrogen adsorption data in the relative pressure (*P*/*P*°) range of 0.06–0.40. The total pore volume was determined from the amount of nitrogen adsorbed at *P*/*P*° = 0.98. The mesopore size distribution was determined by the Barret-Joyner-Halenda (BJH) model applied to desorption data, and the assessment of microporosity was carried out by the t-plot method. For the porosimetry analysis of biochars, a minimum equilibrium interval of 30 s with a maximum relative tolerance of 5% of the targeted pressure and an absolute tolerance of 5 mmHg were used. Before analysis, biochars were activated in situ, by heating at 300 °C, at a rate of 5 °C min^−1^, under a high vacuum (< 10–8 mbar) for 12 h, provided by an oil-sealed mechanical vacuum pump coupled with a high-vacuum system.

The ash content was determined according to the ASTM International D 2866–11 (American Standard Test Method (ASTM), 2018), by incinerating a known amount of biochar (previously dried in an oven at 105 °C for 1 h) in a muffle at 650 °C for 1 h and then weighting again.

For the elemental analysis, aliquots of about 2 mg of finely ground samples, dried at 120 °C for 24 h, were analyzed in triplicate using a FlashEA® 1112 elemental analyzer (Thermo Fisher Scientific, Waltham, MA) equipped with a thermal conductivity detector. The percentage content of oxygen was determined by difference as follows: O(%) = 100 − (C% + H% + N% + S% + ash%).

X-ray diffraction (XRD) was adopted to investigate the occurrence of a graphitic portion in the prepared biochars by using the X-ray diffractometer platform D8 Advance (Bruker, Billerica, MA, USA) equipped with a Bruker LYNXEYE-XE detector. The measurements were performed according to the powder standard diffraction processes.

### Calibration plot

The calibration plots were fitted to a linear model function (*y* = *ax* + *b*) by linear least-squares method, by using Origin Pro 2019b software (Origin Lab Corporation, USA).

The limit of detection (LOD) was calculated according to the equation:

$$\mathrm{LOD}=3.3\cdot(s_{y/x}/S)$$

where *s*_*y/x*_ is the residual standard deviation and *S* is the slope of the calibration plot (calibration sensitivity). The following equation was used for the limit of quantification (LOQ) evaluation:

$$\mathrm{LOQ }= 10 \cdot(s_{y/x}/S)$$

### Anthocyanins

The aglycon structures of the anthocyanins studied in their chloride salt forms, drawn with ChemBioDraw 15.0 (PerkinElmer, MA, USA), are shown in Scheme S1.

Stock solutions of the various anthocyanins were prepared at 4 °C at a concentration of 1 mg mL^−1^ in a methanol/H_2_O mixture, except for myrtillin which was prepared at a concentration of 5 mg mL^−1^ in 0.1 M HCl (aq). The compounds were all stored at 4 °C and kept away from light.

The concentrations present in the mixtures are the following:

- *V. myrtillus* (bilberry): delphinidin 3-glucoside: 500 mg L^−1^, cyanidin 3-galactoside: 300 mg L^−1^, petunidin 3-glucoside: 200 mg L^−1^, peonidin 3-glucoside: 100 mg L^−1^, and malvidin 3-glucoside: 200 mg L^−1^
- *Vaccinium uliginosum* subsp*. gaultherioides* (false bilberry): delphinidin 3-glucoside: 100 mg L^−1^, cyanidin 3-galactoside: 40 mg L^−1^, petunidin 3-glucoside: 125 mg L^−1^, peonidin 3-glucoside: 40 mg L^−1^, and malvidin 3-glucoside: 375 mg L^−1^
- *Fragaria* × *ananassa* (garden strawberry)*:* cyanidin 3-glucoside: 10 mg L^−1^, peonidin 3-glucoside: 1 mg L^−1^, and pelargonidin 3-rutinoside: 30 mg L^−1^

Since the vast majority of anthocyanins contained in the solution is oxidized completely after the first scan, a fresh solution from the stock was prepared for each scan. A final volume of 1000 µL was used for these analyses.

### Berry extraction

Berry extraction was performed as elsewhere reported [32]. Briefly, freeze-dried berry aliquots (500 mg dry weight) were extracted twice with a methanol/H_2_O solution 8/2 (*v/v*) in an ice bath under magnetic stirring.

## Results and discussion

### Physicochemical characterization

Two types of sewage-sludge-biochar (SSB) produced by the same biomass of origin but treated (SSB-T) or untreated (SSB-U) with an acidic solution were used to prepare the SSB–based CPEs. Acid treatments of biochar generally enhance metal removal and modify the porous structure of biochar [34]. These treatments may be beneficial due to enhanced properties but create additional costs and decrease the sustainability of the final material because mineral acids are mainly used. Here, a more environmentally friendly acidic treatment was performed by using an acidic liquid by-product of the gasification of woody waste. The effect of this treatment on the physicochemical characteristics of biochar was studied. Thus, the surface area, the graphitization degree, the pore size distributions, and the elemental analysis were evaluated for both SSBs. Table S1 illustrates the results obtained for the porosimetry analyses (i.e., BET specific surface area, t-plot micropore volume, BJH desorption cumulative mesopore volume, and total pore volume). The mean values of the specific surface area of the two biochars were 319 and 344 m^2^ g^−1^ for SSB-U and SSB-T, respectively. It should be noted that these results were obtained after degassing at 300 °C, a temperature value corresponding to a very small weight loss of the materials during thermogravimetric analysis, as illustrated in Fig. S1. This finding supported the reliability of the results obtained for the evaluation of the surface area data, which are quite high, considering that the biochars were obtained from 30% of biological sludge mixed with wood waste from forest cutting. In fact, even though biochars derived from mixtures of biosolids and vegetal waste are poorly described in the literature, the available values of surface area are lower than those determined in this study [35, 36].

Reversible type II nitrogen adsorption and desorption isotherms were obtained for both SSB-U and SSB-T, with H4-like hysteresis loop [37], as shown for SSB-T in Fig. S2. This behavior could be attributed to the presence of narrow slit-like pores and/or particles with internal voids characterized by irregular shapes and broad size distribution, which is consistent with the heterogeneous characteristics of the feedstock used, i.e., two different kinds of biomass, each one of them and particularly sludge characterized by highly variable compositions. The considerations regarding the structural heterogeneity of the biochars were confirmed by SEM analysis (Fig. S3). In fact, all samples showed a heterogeneous surface morphology without noticeable occurrence of closely bound sludge-wood matrix. Different forms may be observed in all biochars, especially elongated shapes probably derived from the woody material, which mainly consists of cellulose and hemicellulose and simply small formless particles that could have been originated by the sewage sludge [38]. Honeycomb-like shapes have been observed on the surface of biochars after the acid washing, the appearance of which is in agreement with the increase in surface area and total pore volume observed by the physisorption analyses.

Both biochars were mainly mesoporous, since 84% and 62% of the total pore volume of SSB-U and SSB-T, respectively, were in the pore diameter range 2–50 nm, mainly in the low pore width around 4 nm (see the example of SSB-U in Fig. S4). The acidic treatment gave rise to a slight increase in the surface area and total pore volume of the material and an appreciable shift from mesoporosity to macroporosity. These changes are consistent with the low pH value (pH ≈ 2.5) of the acidic solution that may dissolve salts and metals present in the pores, thus increasing their volume. The determination of ash was also consistent with the aforementioned considerations, as its content strongly decreased (Table S2). Moreover, the treatment of biochar affected the content of leachable metal (Table S3). More in detail, As, Hg, and especially Cr content were strongly reduced. In particular, the level of the total Cr content is far below the legal limit accepted for the use of granular activated carbon for the treatment of water intended for human consumption (UNIEN 12915–1).

The acidic treatment was also able to strongly increase the oxygen percentage of the material, which was found to be three times higher in SSB-T than in SSB-U, while the other elements remained quite stable. XRD analysis confirmed the role of acidic washing in the removal of the inorganic fraction, since the several peaks associated with hydroxyapatite and calcite in SSB-U were almost completely absent in SSB-T (Fig. S5). In both materials, XRD also highlighted the occurrence of significant amounts of amorphous carbon, while the crystalline structure represented a minority fraction.

### Electrochemical characterization of SSB-CPEs

**Fig. 1:**
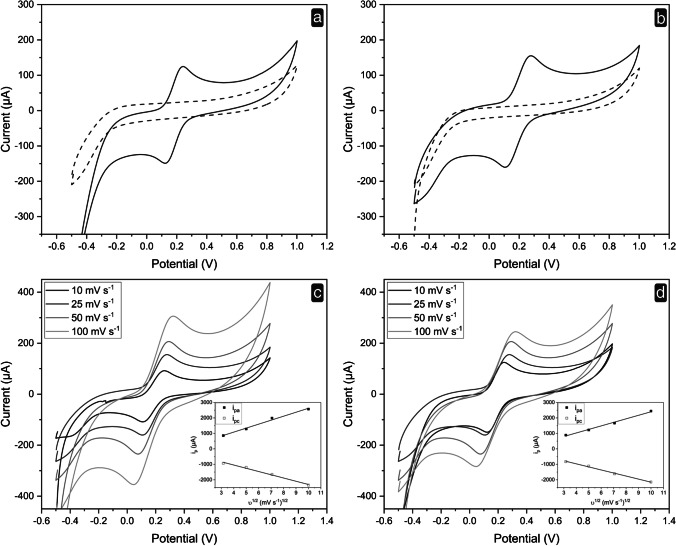
Electrochemical characterization of SSB-CPEs: **a** CV for 0.1 M KCl (dashed line) and 5 mM Fe(CN)_6_^3−/4−^ in 0.1 M KCl (solid line) recorded at SSB-U CPE, scan rate 25 mV s^−1^; **b** CV for 0.1 M KCl (dashed line) and 5 mM Fe(CN)_6_^3−/4−^ in 0.1 M KCl (solid line) recorded at SSB-T CPE, scan rate 25 mV s^−1^; CVs of 5 mM Fe(CN)_6_^3−/4−^ in 0.1 M KCl at different scan rates recorded at **c** SSB-U CPE and **d** SSB-T CPE, whereas the insets show the plots of the CV anodic (*i*_pa_) and cathodic (*i*_pc_) peak currents vs. square root of scan rate (*ʋ*^1/2^)

SSB-U and SSB-T were then used to prepare CPEs. These were characterized for their electrochemical properties. Figure 1a, b reports the CV profiles of SSB-U and SSB-T CPEs, respectively. The measurements were performed in the absence and in the presence of the inner-sphere redox indicator Fe(CN)_6_^3−/4−^ [39], a molecule characterized by an electrochemical behavior that is more sensitive to the chemistry (oxygen-containing functionalities, impurities, and adsorption sites) and structure of the electrode material surface, rather than its electronic density of states.

The CVs at both CPEs do not show any redox signal when recorded in 0.1 M KCl (Fig. 1a, b), and the CV scan line can be ascribed to only a capacitive current in the potential range investigated. This means that the inorganic impurities shown by XRD and trace element analysis did not give rise to a faradic contribution to the current, and the potential range from −1 to +1 V can be used for electroanalytical purposes. In the presence of the inner-sphere redox indicator, a peak-shaped profile is observed. Both electrodes exhibit a quasi-reversible behavior with the hexacyanoferrate (II)/(III) couple (Fig. 1a, b). The electrochemical parameters extracted from the responses of the two types of electrodes are summarized in Table 1.

**Table 1:** Electrochemical parameters obtained from the CV scans recorded at the SSB-U and SSB-T electrodes for an equimolar solution containing 2.5 mM Fe(CN)_6_^3*−*^ and 2.5 mM Fe(CN)_6_^4*−*^ in 0.1 M KCl. The potential was scanned with a scan rate of 25 mV s^*−*1^

	*E*_pa_ (V)	*E*_pc_ (V)	Δ*E* (V)	(*E*_pa_ − *E*_pa/2_) (V)	(*E*_pa_ + *E*_pc_)/2 (V)	*i*_pa_ (μA)	*i*_pc_ (μA)	*i*_pa_/*i*_pc_
SSB-U	0.295 ± 0.014	0.117 ± 0.019	0.178 ± 0.028	0.132	0.206 ± 0.021	151 ± 7	179 ± 8	0.84 ± 0.05
SSB-T	0.271 ± 0.017	0.118 ± 0.017	0.153 ± 0.015	0.104	0.194 ± 0.018	164 ± 9	187 ± 10	0.88 ± 0.06

In all cases, the potential separation between the two peaks (Δ*E*) and the difference in potential between the peak potential (*E*_pa_) corresponding to the anodic peak current, and the potential corresponding to half the peak current (|*E*_pa_ − *E*_pa/2_|) were larger than the theoretical values calculated for a totally reversible redox system involving one electron (i.e., 0.059 V). These data suggest a quasi-reversible behavior, confirmed by the *i*_pa_/*i*_pc_ ratio and already demonstrated for other CPEs [40].

Interestingly, the (*E*_pa_ + *E*_pc_)/2 values (the potential midway between the anodic and cathodic peaks, corresponding to the formal potential) were similar for both CPEs.

The surface area of the CPEs was determined using the Randles–Ševčík equation, as reported in the Supplementary information. The electrochemically active surface area of the CPEs was found to be 0.53 cm^2^ and 0.57 cm^2^ for SSB-T and SSB-U CPEs, respectively. By contrast, the geometrical area of both electrodes is 0.20 cm^2^. The values of the electrochemically active surface area can be explained by considering the data obtained by BET analysis and porosity values.

Furthermore, the linearity of the plots of both anodic and cathodic peak currents, reported versus the square root of the scan rate, confirms the diffusion-controlled electron transfer (Fig. 1c, d).

The electrical properties of SSB materials have also been studied by electrochemical impedance spectroscopy in 0.1 M KCl containing 5 mM Fe(CN)_6_^3−/4−^ in a three-electrode cell configuration. In EIS, a key parameter is the charge transfer resistance (*R*_ct_), given by the semicircle diameter of the Nyquist plot. Both CPEs show small semicircles. In particular, the *R*_ct_ was found to be of 230 ± 11 Ω and 367 ± 7 Ω for the SSB-U CPE and the SSB-T CPE, respectively, thus suggesting good conductivity and electron transfer process between the redox indicator Fe(CN)_6_^3−/4−^ and the SSB-based CPE (Fig. S6).

To better characterize the electroanalytical performances, SSB-U and SSB-T CPEs were challenged with phenolic-containing compounds. As reported in the literature, phenol redox reactions are very sensitive to the condition of a carbon electrode surface [27] and are commonly used for the characterization of the electrode’s performance. Phenolic compounds present different electrochemical behaviors depending on their hydroxyl moieties arrangement. Thus, different phenols such as hydroquinone as a model *ortho*-diphenol, catechol as a model of *para*-diphenol, and resorcinol as *meta* isomer were tested. Furthermore, the behavior of gallic acid (a triphenol) and vanillin (a monophenol) was also evaluated.

Hydroquinone and catechol are well known to undergo reversible oxidation to quinone by the transfer of two electrons and two protons [41]. The reversibility of the hydroquinone and catechol redox processes was confirmed by CV scans (Fig. S7). By contrast, the oxidation product of resorcinol is not thermodynamically stable [41, 42], leading to an irreversible profile in CV (Fig. S7).

**Fig. 2:**
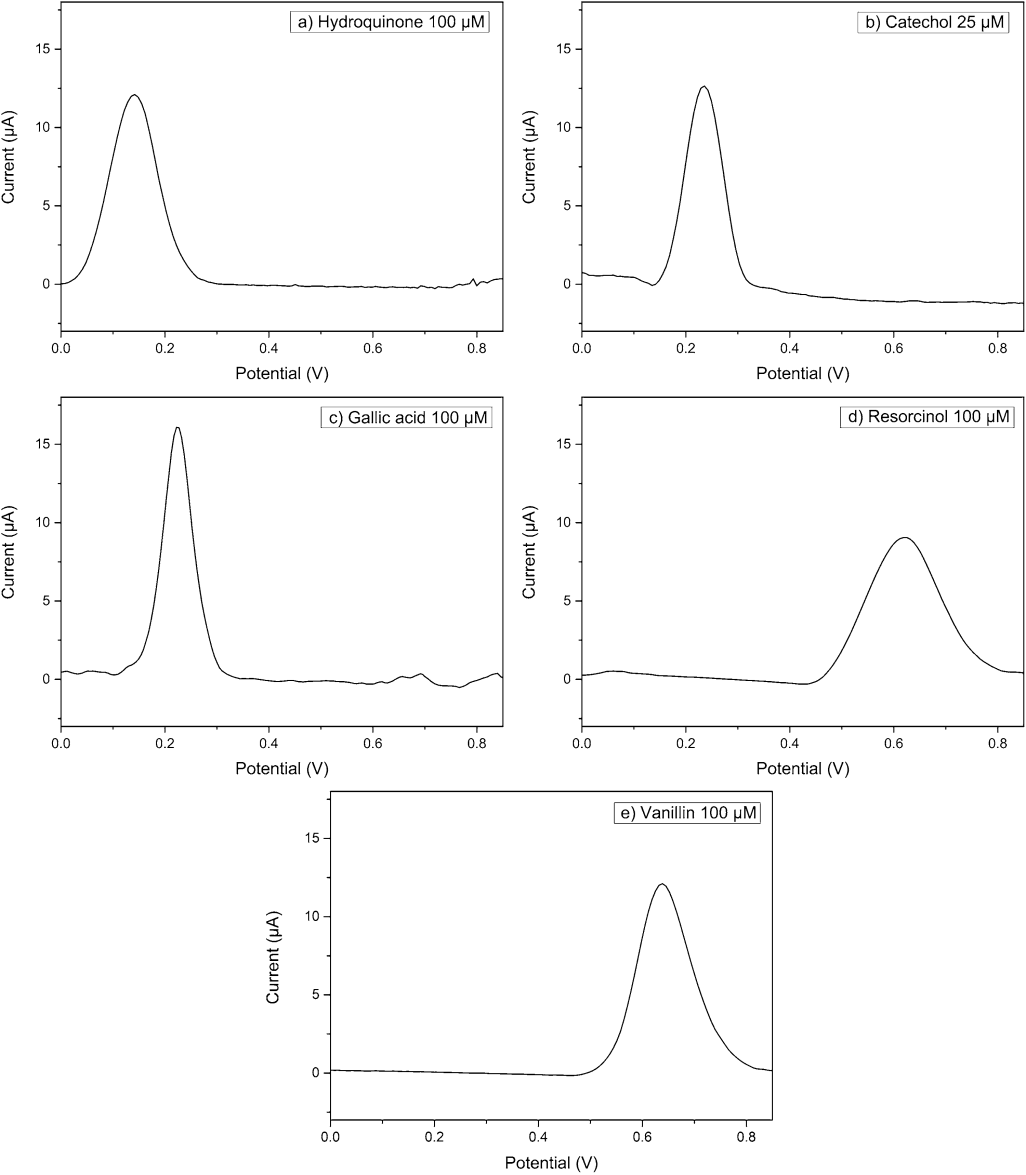
Differential pulse voltammograms recorded in 0.1 M acetic buffer pH 4.75 at SSB-U CPE for **a** 0.1 mM hydroquinone; **b** 0.025 mM cathecol; **c** 0.1 mM gallic acid; **d** 0.1 mM resorcinol; **e** 0.1 mM vanillin; pulse amplitude 90 mV, pulse width 60 ms, and scan rate 30 mV s^−1^

DPV scans of hydroquinone, catechol, gallic acid, and resorcinol in acetic buffer, pH 4.75, are reported in Fig. 2. Furthermore, a DPV analysis of the various compounds was performed at different concentrations and the corresponding details of the calibration plots are reported in Table 2. The DPV electrochemical behavior of catechol and hydroquinone is similar. Indeed, for both compounds, only one anodic peak was obtained at both CPEs (Table 2) and the oxidation potentials are consistent with those reported in the literature for a glassy carbon electrode [42]. As reported in Table 2, resorcinol oxidation occurs at a higher potential value in comparison to hydroquinone and catechol at both CPEs. The higher oxidation potential of resorcinol relative to hydroquinone or catechol is explained by the differences in reactivity of the isomers. The reactivity of the phenolic ring increases when the –OH group is in the *ortho* or *para* position since the highest electron density is located at these sites. Therefore, hydroquinone and catechol, but not resorcinol, have the aromatic ring activated.

**Table 2:** DPV peak potentials, sensitivities, *r*^2^, LODs, and LOQs resulting from calibration curves of the different phenolic compounds

Electrode	Phenolic compound	Peak potential (V)	Sensitivity (μA μM^−1^)	*r*^2^	LOD (μM)	LOQ (μM)
SSB-U	Hydroquinone	0.139 ± 0.005	0.097 ± 0.002	0.997	8	23
	Catechol	0.235 ± 0.009	0.36 ± 0.01	0.991	15	46
	Gallic acid	0.214 ± 0.014	0.171 ± 0.006	0.991	17	52
	Resorcinol	0.629 ± 0.007	0.095 ± 0.004	0.995	12	34
	Vanillin	0.654 ± 0.012	0.102 ± 0.003	0.996	8	25
SSB-T	Hydroquinone	0.132 ± 0.005	0.110 ± 0.004	0.991	12	38
	Catechol	0.228 ± 0.009	0.34 ± 0.01	0.990	15	47
	Gallic acid	0.209 ± 0.015	0.180 ± 0.006	0.991	17	52
	Resorcinol	0.667 ± 0.005	0.121 ± 0.005	0.992	9	28
	Vanillin	0.615 ± 0.006	0.147 ± 0.004	0.993	9	27

Gallic acid, with three –OH moieties, undergoes a reversible oxidation at lower potential values, and its CV (Fig. S7) and DPV profiles (Fig. 2c) are similar to those of catechol. This behavior is obtained at both CPEs. By contrast, vanillin, which can be considered as a monophenol [43], oxidizes at a potential higher than catechol (Fig. 2e).

In conclusion, SSB-CPEs are able to distinguish among the different phenol structures confirming the rule that *para*- and *ortho*-phenol positions oxidize at a lower potential than *meta*-phenol and monophenol.

Moreover, different concentrations of each phenolic compound were analyzed in DPV and a linear behavior in the range 10–150 µM was experimentally found for both CPEs. The calculated detection limits are in the range of those published for other carbon paste electrodes; thus, the sensitivity of these CPEs is in the analytical range of interest for further applications in food sample analysis.

Intra-electrode reproducibility was assessed. For this purpose, 100 µM hydroquinone (HQ) in acetate buffer pH 4.75 was analyzed and a RSD = 8% and RSD = 9% (*n* = 12) for SSB-U and SSB-T, respectively, was observed. Furthermore, inter-electrode reproducibility calculated under the same experimental conditions was found to be RSD = 22% and RSD = 23% (*n* = 12).

The experimental results show that the two CPEs have similar electrochemical properties in terms of resistance, conductivity, and efficiency of the electrochemically active surface toward the detection of molecules with redox properties. However, the physicochemical characterization shows a difference between the two materials in terms of inorganic contaminants such as chromium and arsenic, which are efficiently removed by the acidic treatment. In this regard, it should be remarked that the concentration of each trace element in SSB-T is lower than the value legally accepted for the use of granular activated carbon. Thus, due to this “environmentally friendly” attribute, the SSB-T CPE was used for further experiments.

### Direct electroanalysis of anthocyanins in standard mixtures and extracts

Anthocyanins are one of the major groups of pigments present in plants responsible for the different colors of many flowers, fruits, and vegetables. There are more than 600 anthocyanins in nature, each of them characterized by diverse methoxyl and hydroxyl substituents and by the position, type, and number of the sugar moieties. The substituent groups on the anthocyanin mainly influence reactivity and color by changing the electron distribution on the molecule. Anthocyanins possess a number of pharmacological and nutraceutical properties correlated with their antioxidant activity [44]. The most common anthocyanins found in *Vaccinium myrtillus* and *Fragaria* × *ananassa* are pelargonidin (Pg), peonidin (Pn), petunidin (Pt), malvidin (Mv), cyanidin (Cy), and delphinidin (Dp), the chemical structures of which are reported in Scheme S1. All phenolic groups present on the catechol (in blue in Scheme S1) and the resorcinol moieties of anthocyanins can be electrochemically oxidized.

At pH < 2 the anthocyanin exists primarily in the form of the red or yellow flavylium cation. As the pH is increased, a rapid proton loss occurs, yielding the red or blue quinoidal forms. Then, by hydration of the flavylium cation, a colorless carbinol or pseudobase in equilibrium at a slower rate to the open chalcone is formed. The electrochemical behavior, as well as the antioxidant activity of anthocyanins, is strongly influenced by pH changes, being optimum in the 4.0 < pH < 7.5 range. Thus, acetate buffer at a pH of 4.75 was used for the measurements.

**Fig. 3:**
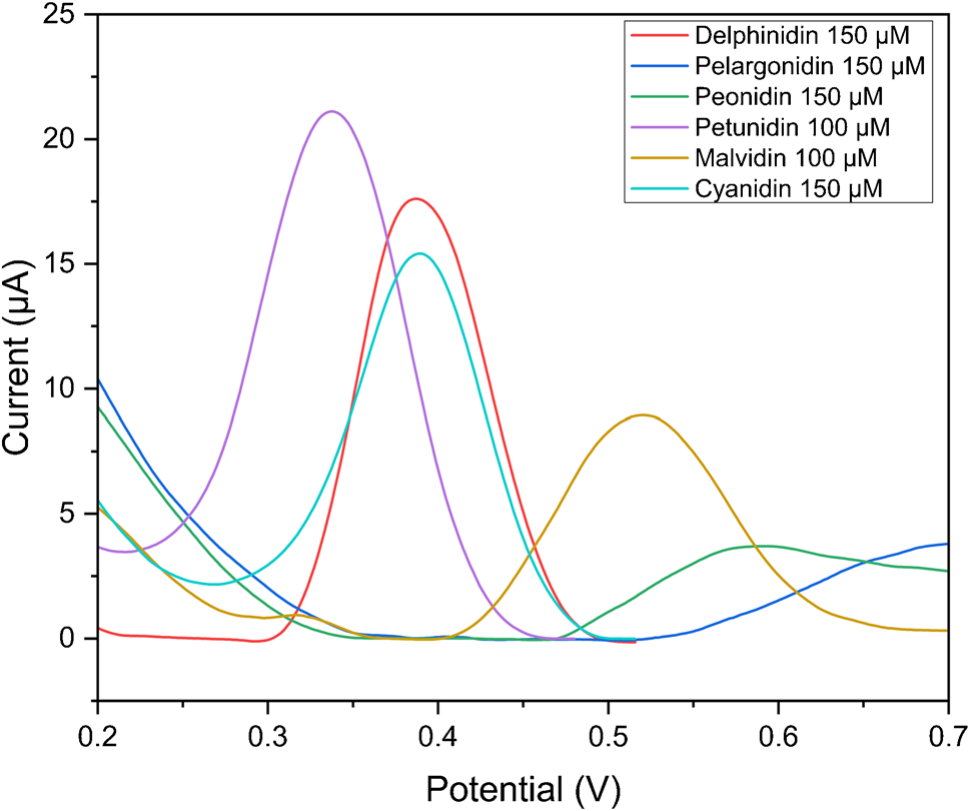
Overlay of the SWV scans for 0.15 mM pelargonidin (Pg), 0.15 mM peonidin (Pn), 0.10 mM petunidin (Pt), 0.10 mM malvidin (Mv), 0.10 mM cyanidin (Cy), and 0.15 mM delphinidin (Dp) in 0.1 mM acetate buffer pH 4.75, recorded at SSB-T CPE; frequency 25 Hz, modulation amplitude 50 mV, potential increment of 2 mV

Square-wave voltammetry (SWV) was used to study the electrochemical behavior of the different anthocyanins and the reversibility of the electron transfer processes (Fig. 3), as proposed by Brett’s group [45]. In SWV, unlike DPV measurements, the current is sampled in both the forward- and the backward-going pulses; thus, oxidation and reduction peaks are obtained in the same experiment (Fig. S8). A strong adsorption of the oxidation products, which caused the fouling of the electrode surface, was also observed. Thus, before the electrochemical measurement, the electrode surface should be gently polished before each new scan by rubbing it for a few seconds on a piece of tissue paper.

As reported in the literature, the oxidation peak related to the resorcinol moiety occurs at high potential values, whereas the diverse hydroxyl (–OH) and methoxyl (–OCH_3_) groups present as substituents of the B-ring determine the diagnostic peaks for the different compounds. The effect of the sugar moieties is not evaluated in this study, even though it is known that they may cause a positive shift in the oxidation potential of the aglycons, due to steric hindrance. However, as reported elsewhere [45], the oxidation peak potential of the hydroxyl groups on the B-ring is not affected by the glucosylation on the A-ring. An overlay of the SWV scans of the net current profile vs*.* the potential for each compound is shown in Fig. 3. Pt, Cy, and Dp show an oxidation peak at +339, +389, and +385 mV, respectively, consistent with the catechol and gallocatechol moieties. As reported in Fig. S8, Pt, Dp, and Cy show a reversible behavior, since both forward and backward currents are present. The fact that the oxidation and reduction peaks occur at the same potential denotes some adsorption of the analyte on the electrode surface. It should be noted that pelargonidin does not show any peak in this potential range and with the reported experimental conditions, whereas the oxidation potential of Pn and Mv is higher than that of Dp.

**Fig. 4:**
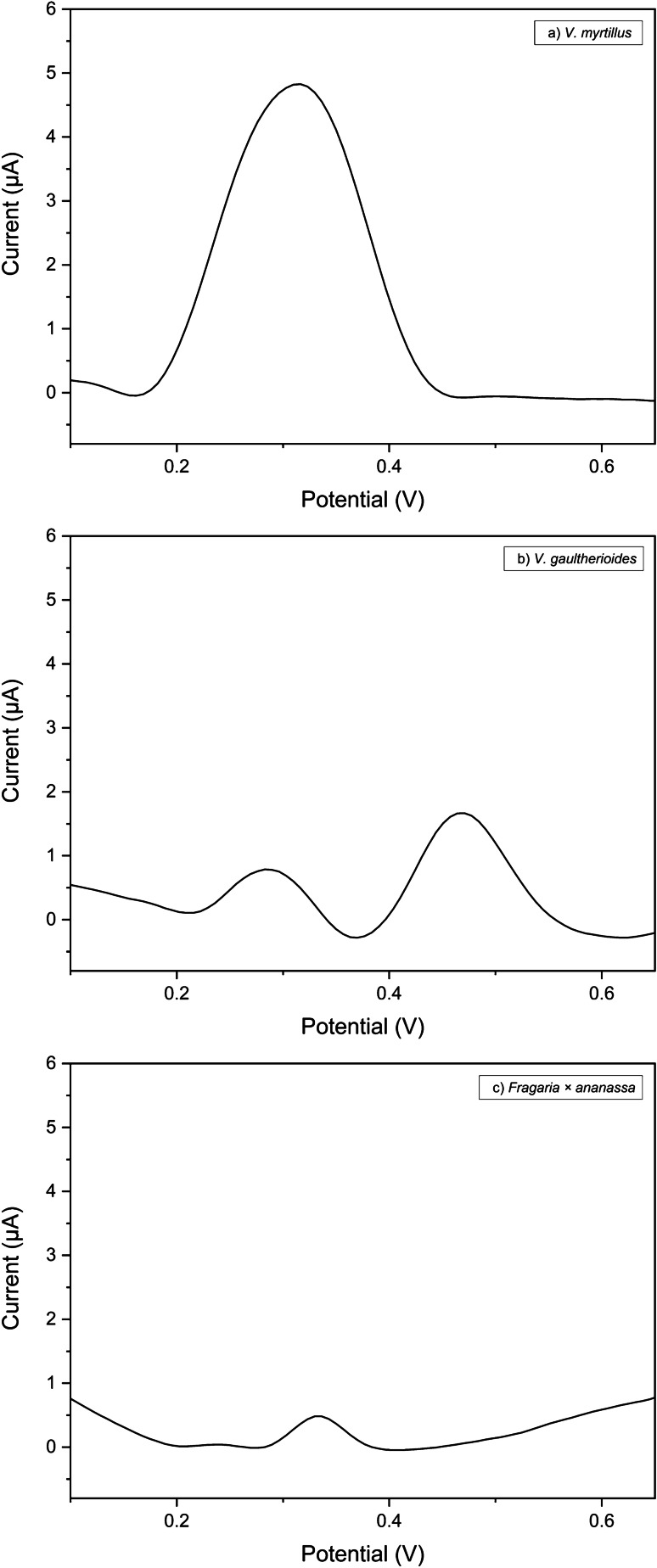
Differential pulse voltammograms for mixtures of anthocyanin standards in 0.1 M acetate buffer pH 4.75 at SSB-T CPE; pulse amplitude 90 mV, pulse width 60 ms, and scan rate 30 mV s^*−*1^. The composition (in terms of type and concentration of anthocyanins) of *V. myrtillus* (dilution ratio 1:10), *V. gaultherioides* (dilution ratio 1:10), and *Fragaria* × *ananassa* (dilution ratio 1:2) is analyzed

Different mixtures, simulating the native anthocyanin composition of bilberry, “false bilberry”, and garden strawberry [32], were prepared using standard solutions and studied by DPV (Fig. 4). The concentrations present in such mixtures are reported in the “Materials and methods” section. Notably, the electrochemical fingerprints of these standard mixtures are very different. As shown, only a broad peak is observed centered at +318 mV for the bilberry (Fig. 4a), whereas two distinct peaks were observed for the “false bilberry” (Fig. 4b), centered at +283 and +464 mV, respectively. Dp, Cy, and Pt are more abundant in the bilberry than in the “false bilberry”, thus explaining the higher peak at +318 mV in Fig. 4a. On the other hand, the peak at +464 mV observed in the DPV profile of the “false bilberry” (Fig. 4b) can be explained by the presence of an almost two-fold higher amount of Mv compared to bilberry. Interestingly, as visible in Fig. 4a, b, a rapid electrochemical evaluation allows noticing the clear difference in the DPV profiles of the two species, which reflect their different composition inanthocyanins. By contrast, it is very difficult to distinguish between *V. myrtillus* and *V. gaultherioides* in nature, since their fruits present similar aspects (for example, in terms of color and shape) and grow in the same zones.

**Fig. 5:**
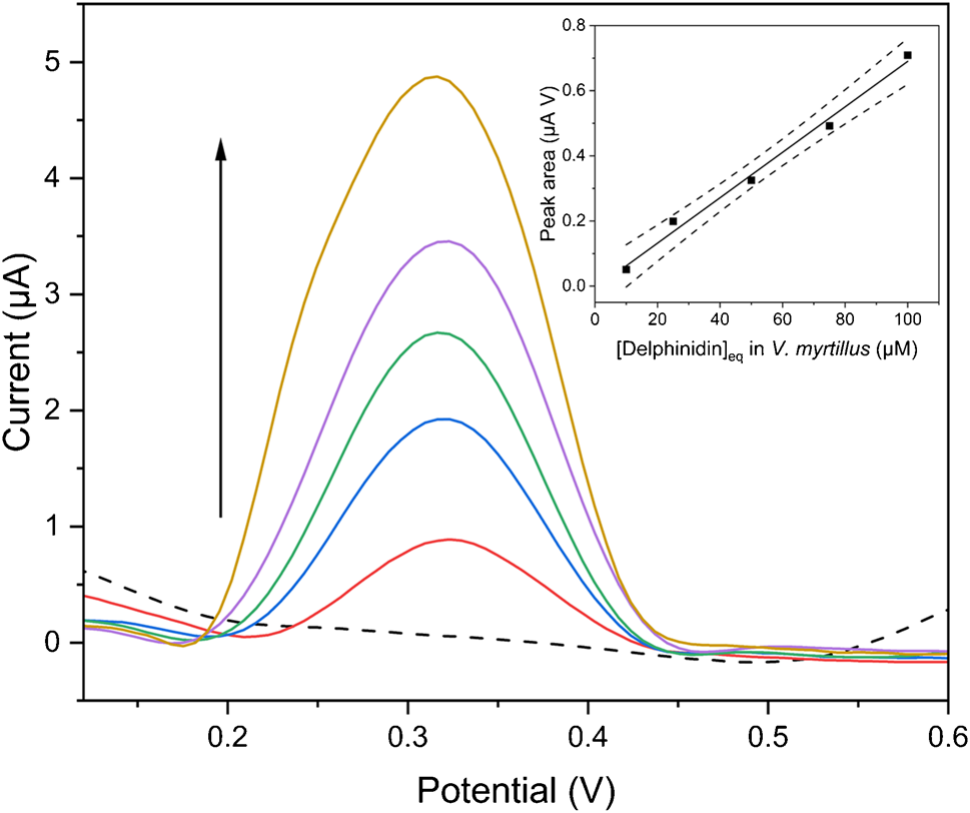
Differential pulse voltammograms of the standard mixtures of *V. myrtillus* at different concentrations of delphinidin (Dp), recorded at SSB-T CPE. Solution containing 10, 25, 50, 75, and 100 µM Dp in 0.1 M acetate buffer pH 4.75; the DPV scan of 0.1 M acetate buffer pH 4.75 is also reported (dashed line); inset: linear fitting of current peak area values versus the concentration of Dp in solution, obtained by diluting the standard mixture of *V. myrtillus*; pulse amplitude 90 mV, pulse width 60 ms, and scan rate 30 mV s^*−*1^

Figure 5 illustrates the DPV measurements on different dilutions of the standard bilberry mixture. A correlation between peak areas and anthocyanins content is observed. In addition, the current peak area values plotted vs. dilution ratios (reported as Dp equivalent concentrations) show a linear behavior. Thus, the response of the CPE is consistent with the changes in anthocyanin concentration in the standard mixture.

In the case of the garden strawberry (Fig. 4c), only a small peak at around +330 mV was observed. This peak is lower than those obtained with the bilberry mixture, considering also the dilution factor. Indeed, this is in agreement with the lower amount of anthocyanins present in the strawberry. The peak at +330 mV can be mainly ascribed to the presence of Cy.

**Fig. 6:**
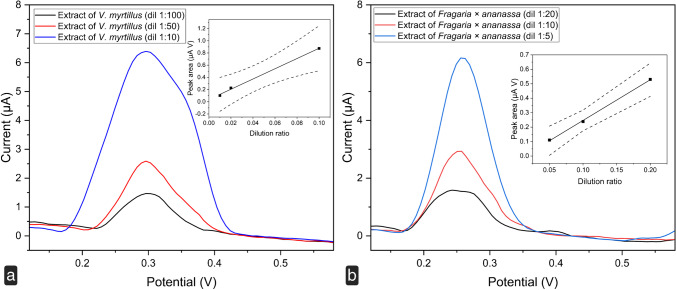
Differential pulse voltammograms of methanolic extracts of **a** *V. myrtillus* and **b** *Fragaria* × *ananassa*, recorded at different dilution factors in 0.1 M acetate buffer, pH 4.75, at SSB-T CPE. Potential scan initiated at 0 V in the positive direction; pulse amplitude 90 mV, pulse width 60 ms, and scan rate 30 mV s^*−*1^

After the analysis of the mixtures prepared with standard solutions to simulate the anthocyanin fractions of *V. myrtillus* and *F.* × *ananassa*, real extracts of the berries were considered. Figure 6a, b shows the electrochemical fingerprints of the extracts of bilberry and garden strawberry obtained by DPV. Different dilutions of the extracts in 0.1 M acetate buffer pH 4.75 were analyzed. As expected, the voltammetric profile of the extracts is slightly different in comparison to the corresponding standard mixture. The fingerprint of *V. myrtillus* shows a broad peak with a maximum centered at +310 ± 13 mV whereas the fingerprint of *F.* × *ananassa* shows a peak centered a +255 ± 11 mV. These oxidation signals are due to anthocyanins, although other electroactive molecules therein present can contribute. As already observed for the standard mixtures, the response of the CPE is consistent with the changes in the concentration of anthocyanins in the extracts. For both extracts, a linear correlation with the dilution ratio was observed. This behavior paves the way for possible future applications of the sensor for the quantitative estimation of antioxidant activity and total phenolic content of berry fruits of nutraceutical interest.

## Conclusions

For the first time, the possibility of using biochar from sewage sludge for electroanalytical applications was demonstrated. Considering the biomass of origin (municipal and industrial sewage sludge), a chemical treatment was considered important in order to obtain a cleaner and safer product with a very low level of inorganic contaminants and heavy metals. Accordingly, an acidic treatment for the removal of inorganic contaminants from the biochar was performed. The biochar was then used to prepare SSB-CPEs. These CPEs were applied to the analysis of various (poly)phenolic compounds, including several anthocyanins, at concentration levels of interest for agri-food applications. Finally, the possibility of using the SSB-CPEs to distinguish different berries from their electrochemical fingerprints was shown. The identification of the different fruits by their electrochemical behavior can be useful for the evaluation of compositional fraud in food and/or supplements and/or pharmaceuticals. In the analysis of the extracts, the concentration-dependent signal paves the way for a possible future quantitative estimation of antioxidant activity and total phenolic content. Thus, the obtained results show that sewage sludge–derived biochar can be a promising material for electroanalytical sensor development.

## Supplementary Information

Below is the link to the electronic supplementary material.

Supplementary file1 (PDF 2.14MB)

## Data Availability

The datasets generated and/or analyzed during the current study are available from the corresponding author on reasonable request.
