# Supplementary material for: Electrochemical sensors based on sewage sludge–derived biochar for the analysis of anthocyanins in berry fruits
Source: Anal Bioanal Chem. 2022 Apr 26;414(21):6295–307. doi: 10.1007/s00216-022-04062-y (PMC9372006; doi:10.1007/s00216-022-04062-y)
Supplement: Supplementary file 1 — Supplementary file1 (PDF 2.14MB) [file 216_2022_4062_MOESM1_ESM.pdf]

Electrochemical sensors based on sewage sludge derived biochar for the analysis of anthocyanins in berry fruits

Analytical and Bioanalytical Chemistry

Patrick Severin Sfragano, Serena Laschi, Lapo Renai, Michelangelo Fichera, Massimo Del Bubba*, Ilaria Palchetti*

Dipartimento di Chimica Ugo Schiff, Università degli studi di Firenze, Via della Lastruccia 3, 50019 Sesto Fiorentino, Italy

ilaria.palchetti@unifi.it, massimo.delbubba@unifi.it

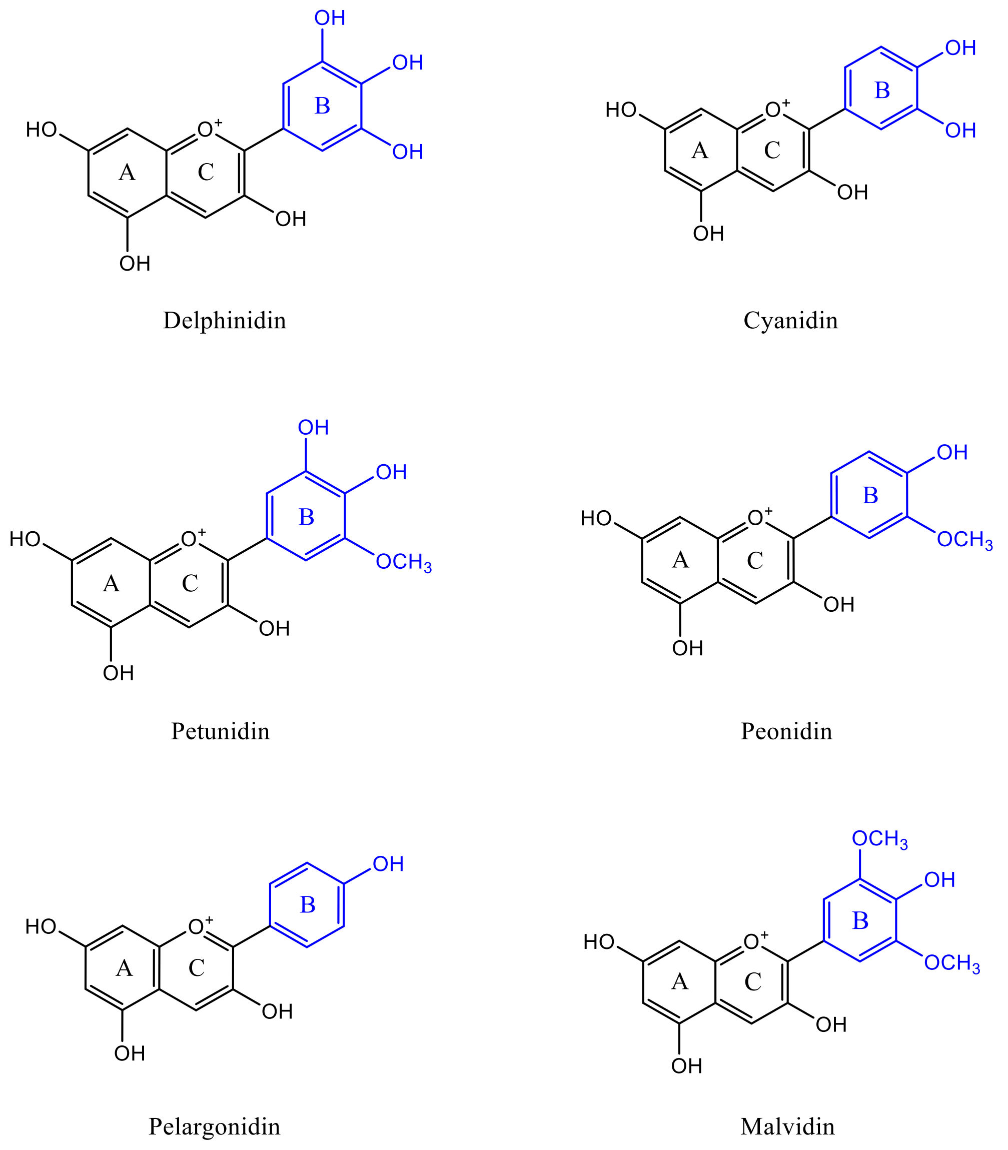

Scheme S1 Aglycon structures of the anthocyanins analyzed throughout this study; the corresponding molecular weight is reported in brackets: delphinidin 3-glucoside (465.4 g mol^{-1}), peonidin 3-arabinoside (433.4 g mol^{-1}); malvidin 3,5-diglucoside (655.6 g mol^{-1}); pelargonidin 3-rutinoside (579.5 g mol^{-1}); cyanidin 3-galactoside (449.4 g mol^{-1}); petunidin 3-glucoside (479.4 g mol^{-1}).

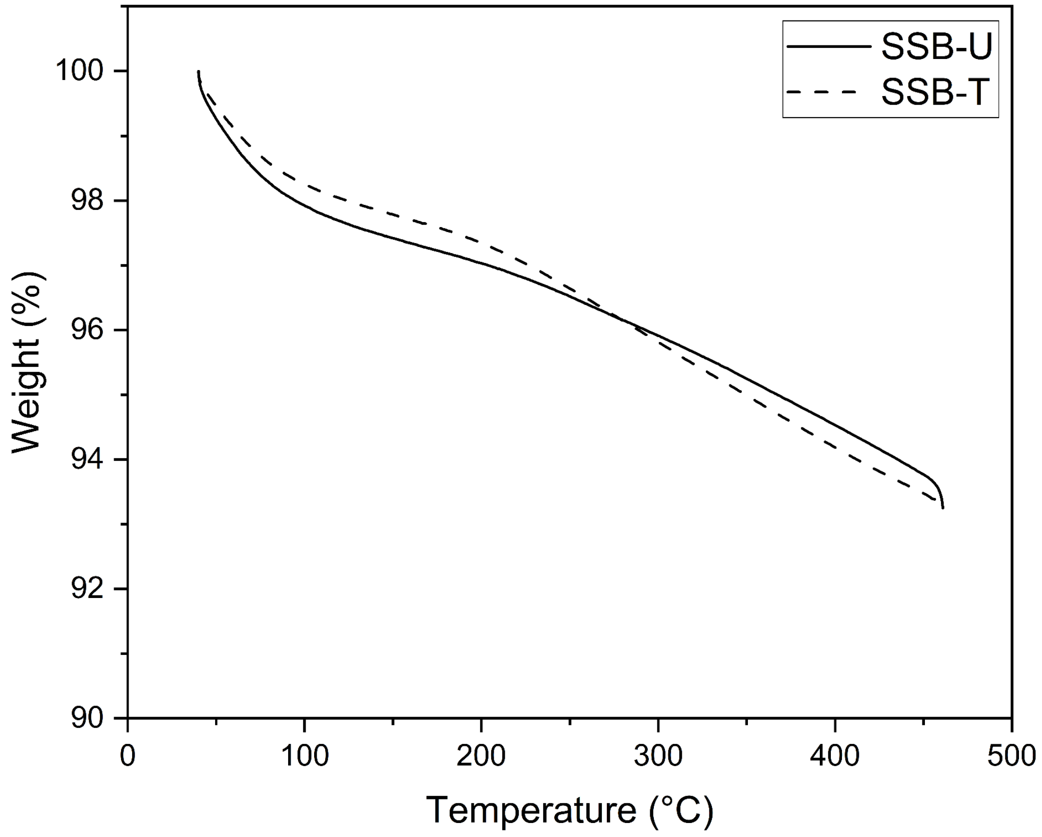

Figure S1 Thermogravimetric analysis of the untreated biochar (SSB-U) and the washing treated biochar (SSB-T) performed from 40 °C to 450 °C at 10 °C min^{-1} under nitrogen flow of 100 mL min^{-1}.

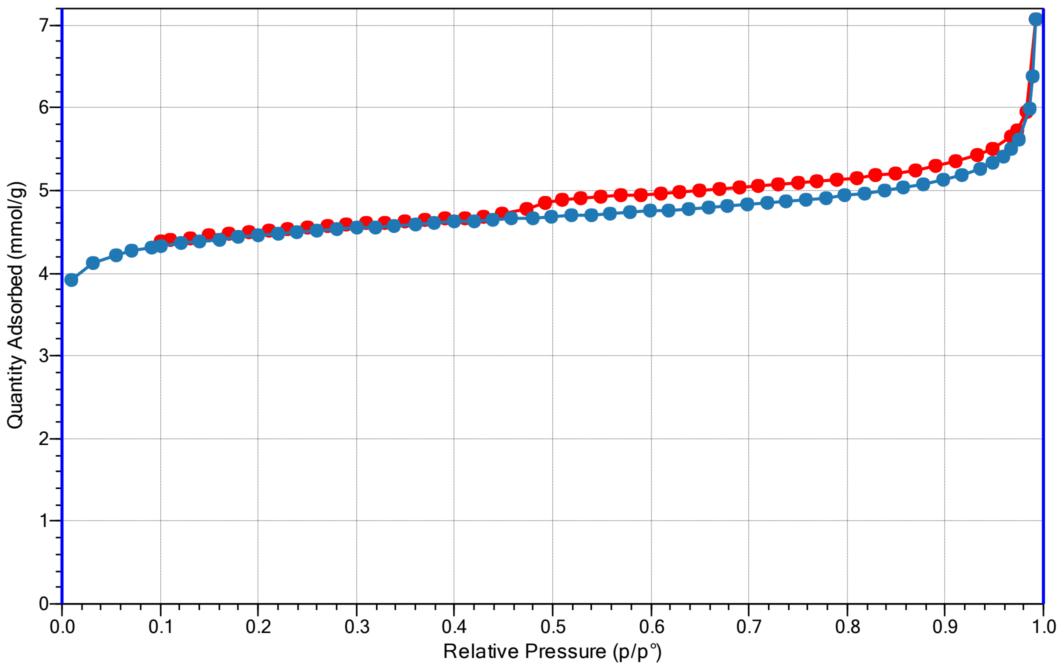

Figure S2 Nitrogen adsorption (blue color) and desorption (red color) isotherms for SSB-T.

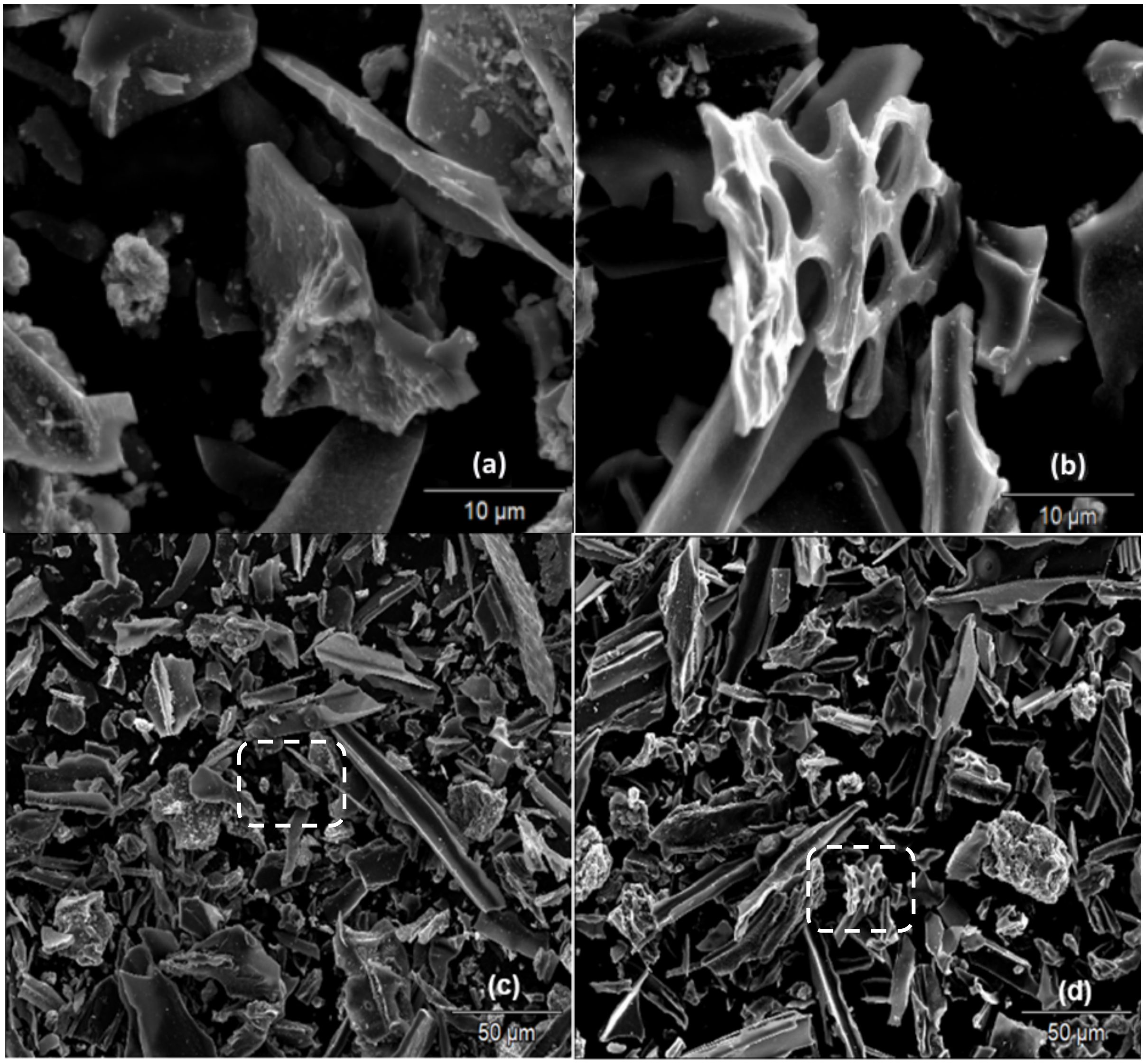

Figure S3 SEM images of SSB-U (panels “a” and “c”) and SSB-T (panels “b” and “d”) taken at different magnification. Dottex boxes identify the regions underwent to magnification.

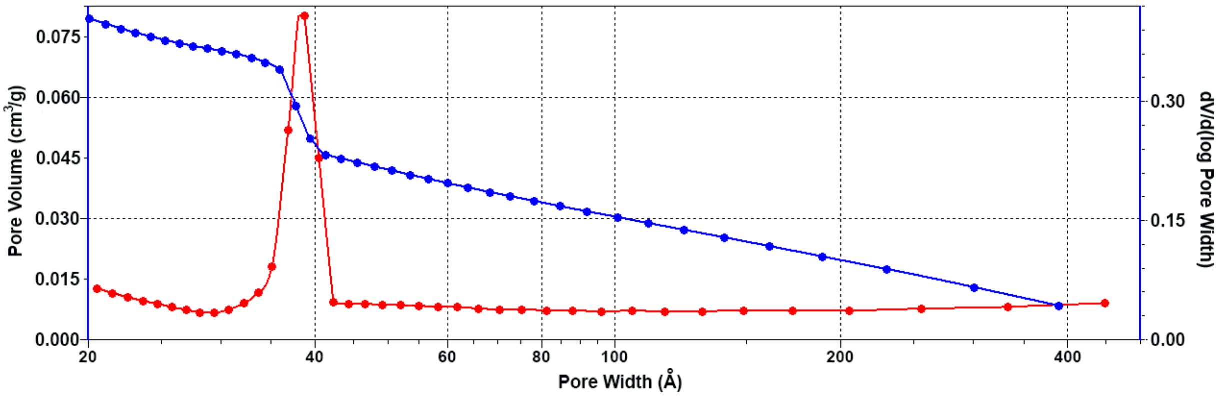

Figure S4 Cumulative pore area of mesopores (blue line) and pore size distribution (red line) calculated by the BJH desorption method for SSB-U.

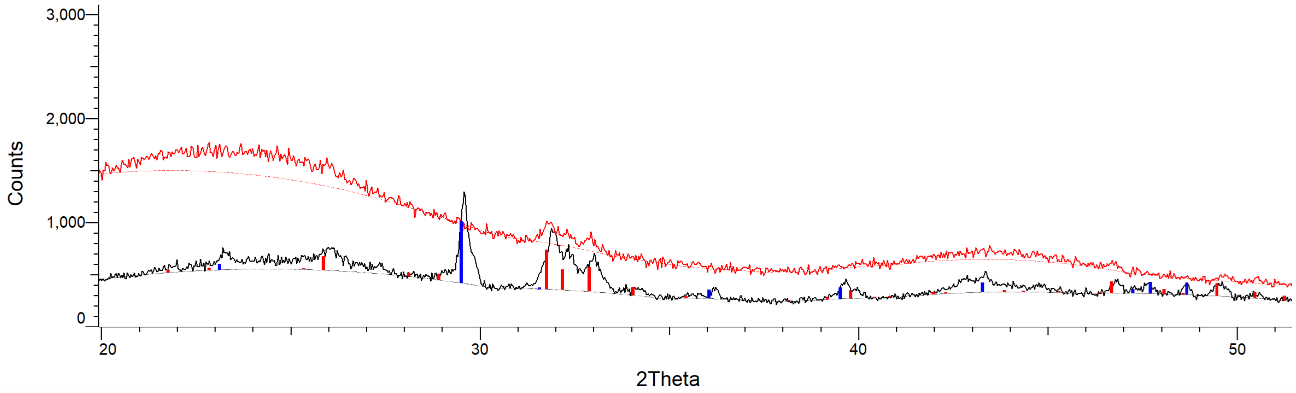

Figure S5 X-ray diffraction analysis of the untreated biochar (SSB-U) and the washed biochar (SSB-T). Black and red traces refer to SSB-U and SSB-T, respectively. Peaks marked with red and blue bars on the SSB-U trace were identified as hydroxyapatite and calcite, respectively, based on the international database PDF4+2021.

Table S1 Specific surface area (n=3) and pore volume distribution of the untreated biochar (SSB-U) and the washing treated biochar (SSB-T).

**Biochar**	**SSB-U**	**SSB-T**
Specific surface area (m^2 g^{-1})	319 (12)	344 (23)
Micropores total volume (cm^3 g^{-1})	0.0052	0.0047
Mesopores total volume (cm^3 g^{-1})	0.087	0.068
Total volume (cm^3 g^{-1})	0.104	0.110

Table S2 Ash content and elemental composition of the untreated biochar (SSB-U) and the washing treated biochar (SSB-T) presented as mean (n = 3) and standard deviation (in brackets).

**Biochar**	**Ash**	**C (%)**	**H (%)**	**N (%)**	**S (%)**	**O (%)**
SSB-U	26.5 (0.7)	63.3 (0.2)	0.8 (0.5)	0.9 (0.1)	<0.05[(*)]	8 (1)
SSB-T	11.1 (0.7)	65.0 (0.9)	1.0 (0.1)	<0.05[(*)]	<0.05[(*)]	23 (2)

[(*)] Detection limit

Table S3 Concentrations (μg kg^{-1}) of selected metals in the untreated biochar (SSB-U) and the washing treated biochar (SSB-T).

**Biochar**	**As**	**Cd**	**Cr**	**Hg**	**Ni**	**Pb**	**Sb**	**Se**
SSB-U	2.6	< 0.05	69.9	0.3	< 0.05	< 0.05	0.3	0.03
SSB-T	0.7	< 0.05	1.8	< 0.05	2.3	1.1	1.7	0.09

Electrochemical surface area evaluation

The surface area of the SSB-T and SSB-U CPEs was determined using the Randles–Ševčík equation [1]

$$i_p = 2.687 \cdot 10^5 \cdot n^{3/2} \cdot A \cdot D^{1/2} \cdot v^{1/2} \cdot C \qquad \textbf{[Eq. S1]}$$

where: i_p is the peak current in A, n is the number of electrons involved in the redox event, A is the area of the electrode in cm^2, D is the diffusion coefficient in $cm^2\ s^{-1}$, v is the sweep rate in $V\ s^{-1}$, and C is the concentration of electroactive species in $mol\ cm^{-3}$.

The value of D_{ox} is the one determined by Konopka and McDuffie [2], that is D_{ox}: $7.26 \cdot 10^{-6}\ cm^2\ s^{-1}$. The concentration used is $2.50 \cdot 10^{-6}\ mol\ cm^{-3}$.

References

1. Trasatti S, Petrii OA (1991) Real surface area measurements in electrochemistry. Pure Appl Chem 63:711–734 . https://doi.org/doi:10.1351/pac199163050711
2. Konopka SJ, McDuffie B (1970) Diffusion coefficients of ferri- and ferrocyanide ions in aqueous media, using twin-electrode thin-layer electrochemistry. Anal Chem 42:1741–1746 . https://doi.org/10.1021/ac50160a042

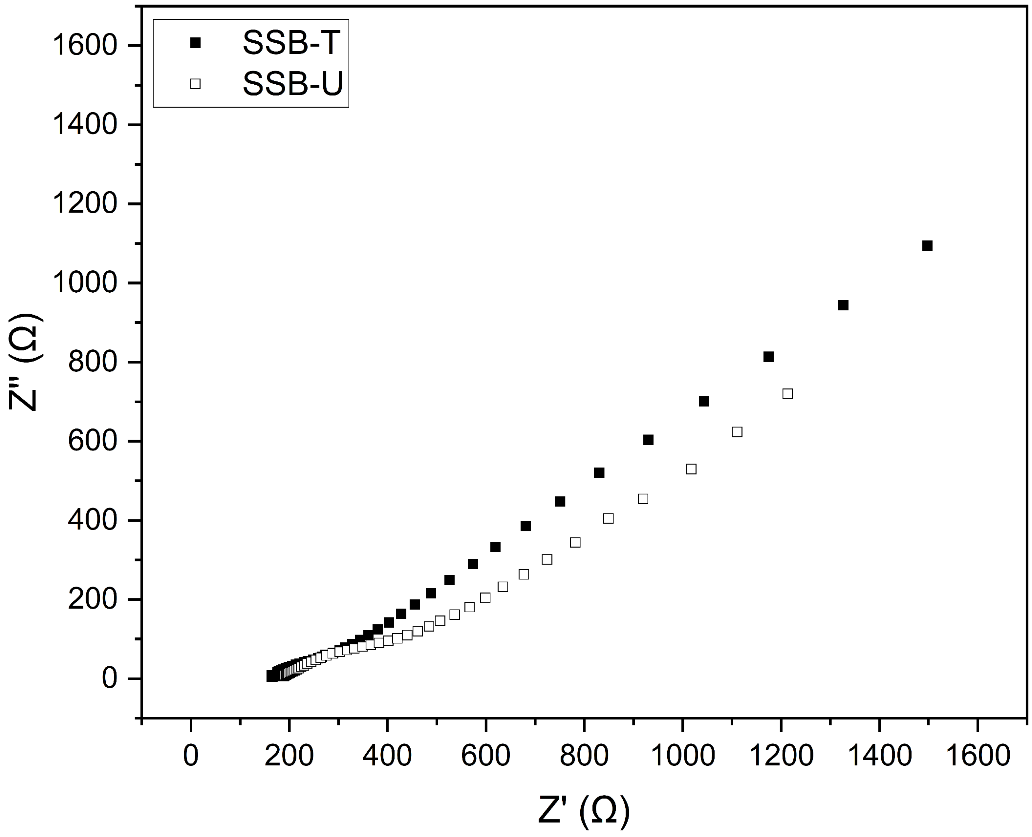

Figure S6 EIS spectra (Nyquist plots) recorded for 5 mM $Fe(CN)_6^{3-/4-}$ in 0.1 M KCl at SSB-U and SSB-T CPEs; EIS parameters are reported in Materials and methods.

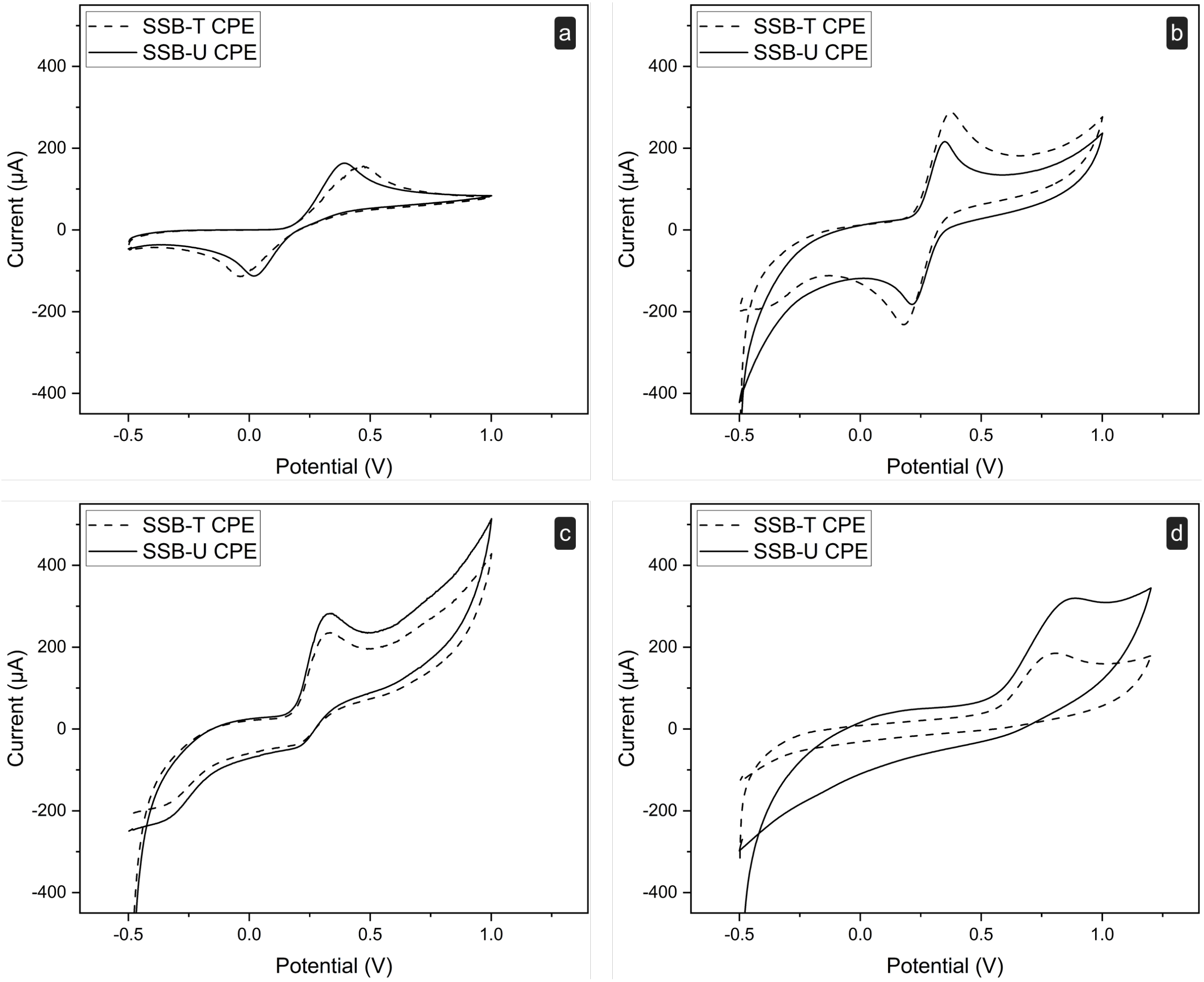

Figure S7 CV scans for **a)** 5 mM hydroquinone in 0.1 M acetic buffer pH 4.7; **b)** 5 mM catechol in 0.1 M acetate buffer pH 4.7; **c)** 5 mM gallic acid in 0.1 M acetic buffer pH 4.7; **d)** 5 mM resorcinol in 0.1 M acetic buffer pH 4.7, recorded at SSB-U and SSB-T CPEs, respectively. Scan rate 25 mV s^{-1}

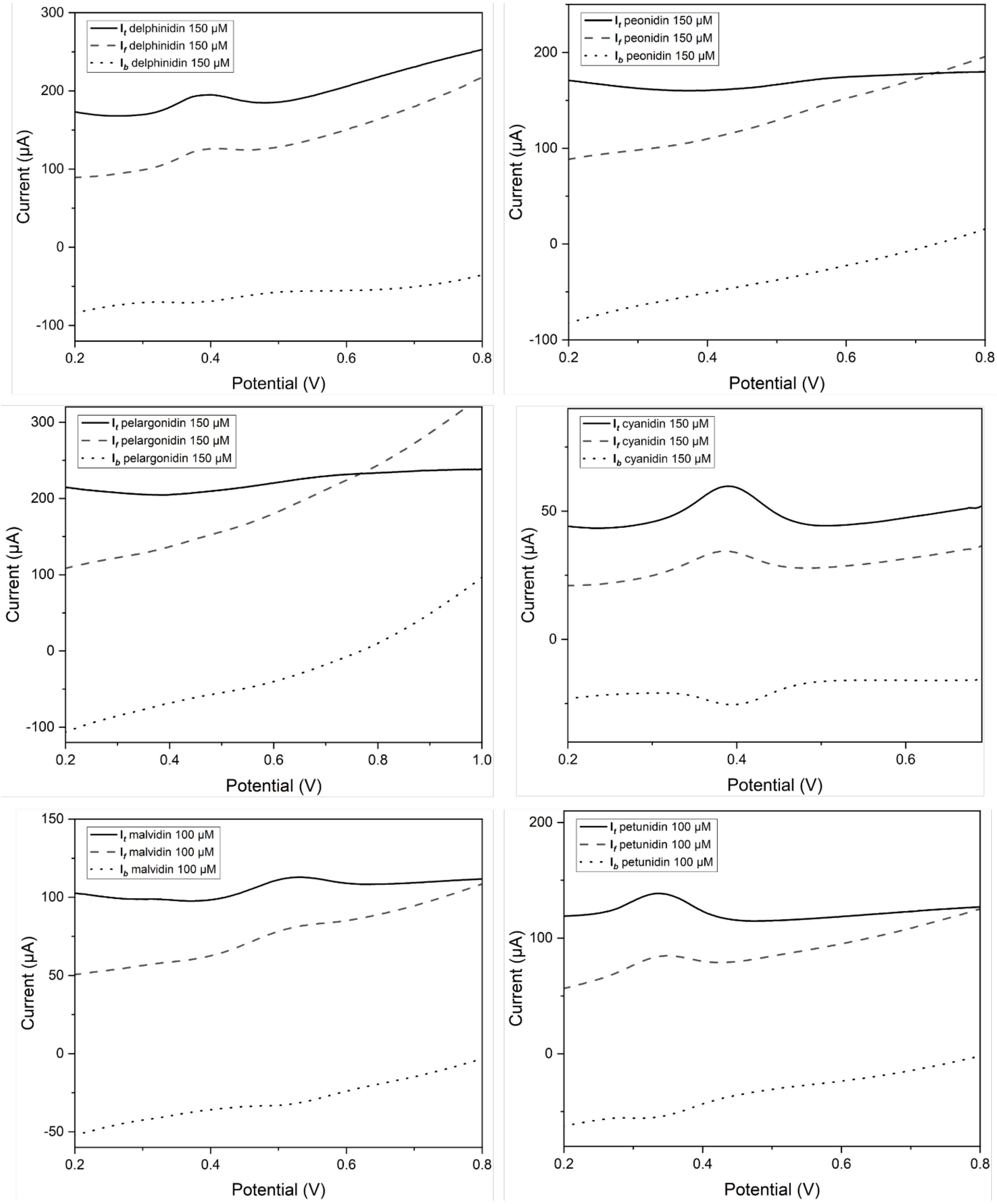

Figure S8 Square wave voltammetry scans for 0.15 mM delphinidin (Dp), 0.15 mM peonidin (Pn), 0.15 mM pelargonidin (Pg), 0.15 mM cyanidin (Cy), 0.10 mM malvidin (Mv) and 0.10 mM petunidin (Pt) in 0.1 mM acetate buffer pH 4.75 recorded at SSB-T CPE; Frequency 25 Hz, amplitude 50 mV. I_t: total current, I_f: forward current, I_b: backward current.
